# Pancreatic β-cell regeneration: advances in understanding the genes and signaling pathways involved

**DOI:** 10.1186/s13073-017-0437-x

**Published:** 2017-05-16

**Authors:** Solomon Afelik, Meritxell Rovira

**Affiliations:** 1 Division of Transplantation, Department of Surgery, University of Illinois at Chicago, 840 South Wood Street, CSB 920 (Rm 502), Chicago, IL 60612 USA; 2 Center of Regenerative Medicine in Barcelona (CMRB), Dr. Aiguader 88, 08003 Barcelona, Spain

## Abstract

Recent advances in β-cell regeneration in vivo are providing insights into the mechanisms involved in the conversion of distinct pancreatic cell lineages into β cells. These mechanisms mostly involve reactivation of the gene encoding the pancreatic endocrine cell-specifying transcription factor neurogenin-3.

## The promise of pancreatic β-cell regeneration

Diabetes mellitus is a metabolic disorder characterized by dysfunction, loss, or insufficient mass of β cells. The main function of β cells is to produce and secrete insulin, the hormone responsible for the regulation of blood glucose levels. Type 1 diabetes (T1D) results from autoimmune destruction of β cells, while type 2 diabetes (T2D) mostly results from β-cell dysfunction or peripheral tissue resistance to insulin, often culminating in β-cell death. Thus, both forms of diabetes can benefit from restoration of β-cell mass. Currently, islet transplantation is the only way to provide new β cells to diabetic patients, but the scarcity of compatible cadaveric donors makes this approach available to only few patients; moreover, it requires lifelong immune suppression [1].

Great progress has been made in the field of in vitro differentiation of human embryonic and induced pluripotent stem cells toward insulin-producing cells, including the generation of stem cell-derived β cells from patients with T1D, leading to an ongoing clinical trial to assess their safety in humans [2]. Recent advances in studies with animal models are also beginning to provide compelling evidence to suggest in vivo β-cell regeneration as a viable alternative future approach to restoring β-cell mass in diabetic patients. These advances have been based on previous studies demonstrating the ability of particular genes and pathways to induce β-cell neogenesis in vivo from differentiated adult pancreatic lineages such as acinar cells, ductal or centroacinar cells, and other endocrine subtypes [1]. It is worth noting that in early development all pancreatic lineages, including acinar cells (which produce digestive enzymes), duct cells (which produce bicarbonate-containing secretions to convey acinar-derived enzymes into the duodenum), and all the pancreatic endocrine subtypes, differentiate from a common pool of multipotent pancreatic progenitor cells (MPCs). MPCs become patterned into acinar progenitor cells and bi-potent ductal/endocrine progenitor cells. The activation of *Ngn3* in this pool of bi-potent progenitors promotes the differentiation of all endocrine cells, while Ngn3-negative bi-potent progenitors mature as ductal cells.

## β-cell regeneration from pancreatic acinar, ductal, or β-cell lineages requires *Ngn3* activation

**Fig. 1:**
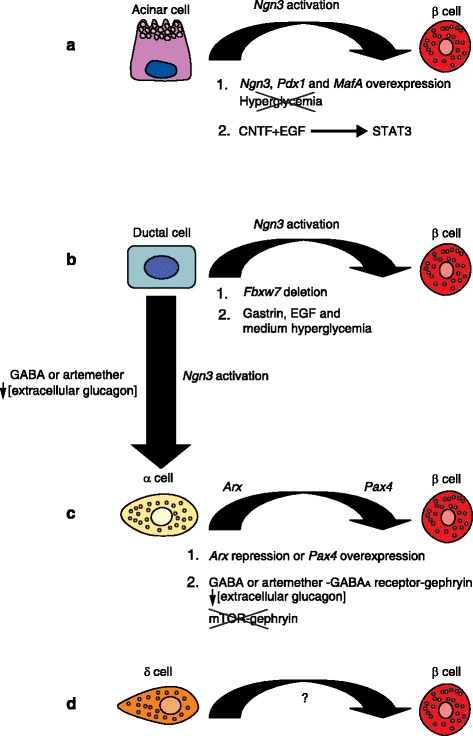
Illustration of cellular plasticity between the different pancreatic lineages and the signaling pathways involved in pancreatic β-cell regeneration. In the regenerative scenarios—acinar to β cell, ductal to β cell, and α to β cell—a transition state involving *Ngn3* activation is necessary for the reprogramming to occur, with the exception of δ to β cell conversion (see [3] and main text for further details). **a** Acinar cells can be converted into β cells in vivo by overexpression of *Ngn3*, *Pdx1*, and *MafA* in mice (1). Hyperglycemia can inhibit (shown by a *cross*) in vivo reprogramming of acinar cells by *Pdx1, Ngn3*, and *MafA*. Furthermore, acinar cells can become β cells in vitro and in vivo after exposure to ciliary neurotrophic factor (*CNTF*) and epidermal growth factor (*EGF*) (2); this regenerative event is dependent on PKA/STAT3 signaling-mediated *Ngn3* activation. **b** Ductal cells can be reprogrammed into β cells in vivo after genetic deletion of the ubiquitin ligase *Fbxw7* (1). Such reprogramming can also be induced in vivo in mice after treatment with gastrin, EGF, and medium hyperglycemia (2). Interestingly, this ductal to β cell conversion requires a progenitor-like intermediate state that involves *Ngn3* activation. **c** Similarly, α cells can be converted into β cells by ectopic activation of *Pax4* in α cells in the adult pancreas, either by transgenic overexpression of *Pax4* or indirectly by suppressing *Arx* (1). In a recent report, it was shown that treatment with an artemisinin (for example, artemether) [9] or long-term administration of gamma-amino butyric acid (*GABA*) [8] results in robust conversion of α cells to β cells in vitro and in vivo (2)*.* Both compounds act through the GABA_A_ receptor; artemether treatment increases gephryin protein levels, which potentiates GABA_A_ receptor signaling while inhibiting mTOR signaling (shown by a *cross*). Strikingly, both artemisinin and GABA treatments inhibit Arx function or expression, leading to induction of *Pax4* expression, concomitantly inhibiting glucagon secretion and inducing insulin expression. Although the in vivo α to β switch in itself is independent of *Ngn3*, the conversion of endogenous α to β cells appears to trigger α-cell neogenesis from ductal cells by reactivation of *Ngn3* in GABA-treated pancreas. **d** Finally, δ cells can be reprogrammed into β cells in early puberty, although this conversion is independent of *Ngn3* activation

Various pancreatic regeneration models have been developed in both mice and rats to study β-cell regeneration in vivo. These models have been used successfully to reprogram several pancreatic lineages into β cells by genetic modifications of key transcription factors, highlighting the prominent role of such genes in β-cell regeneration. These are often genes involved in β-cell development during embryogenesis. Reactivation of the embryonic pancreatic endocrine cell-specifying gene *Ngn3* appears to be critical for sustained β-cell regeneration in vivo in most β-cell regeneration models reported so far [3] (Fig. 1).

While in vivo genetic manipulations are useful for experimentation, they are not feasible for therapy in humans. To translate these studies into therapy, it is of paramount importance to identify signaling pathways or small molecules that can specifically target key β-cell reprogramming genes. Recently, several breakthrough papers have described specific signaling pathways that induce β-cell regeneration from differentiated pancreatic lineages in vivo.

Notably, Baeyens and colleagues [4] translated knowledge gained from in vitro studies to an in vivo mouse model of β-cell regeneration. This study elegantly showed that treating alloxan-induced diabetic mice with epidermal growth factor (EGF) and ciliary neurotrophic factor (CNTF) was sufficient to stimulate the conversion of acinar cells into β cells. Moreover, they provided evidence that this regenerative capacity is dependent on protein kinase A/signal transducer and activator of transcription 3 (PKA/STAT3) signaling, mediating *Ngn3* activation. Interestingly, a subsequent study has illustrated how hyperglycemia can inhibit in vivo reprogramming of acinar cells by the transcription factors *Pdx1*, *Ngn3*, and *MafA* [5], highlighting the significant role of glucose levels in the plasticity and β-cell regenerative potential of the exocrine pancreas. Altogether, these studies provide critical knowledge for β-cell regeneration from pancreatic acinar cells in vivo, with important therapeutic implications given their vast numbers in the pancreas.

Besides regenerating β cells from pancreatic acinar cells, there have been recent advances in reprogramming β cells from pancreatic ducts in vivo. Zhang and co-workers [6] recently described the effectiveness of long-term administration of a low dose of the hormone gastrin (which is produced by the stomach and stimulates the release of gastric acid that helps to break down and digest food) and epidermal growth factor (EGF) under conditions of medium levels of hyperglycemia to reprogram Sox9^+^ ductal cells into β cells in an alloxan-induced diabetic mouse model. In addition, a recent report demonstrated the effect of pro-inflammatory cytokines in inducing β-cell neogenesis in adult mouse duct cells by activating *Ngn3* through STAT3 phosphorylation [7], similar to the requirement of STAT3 signaling for acinar to β-cell regeneration reported by Baeyens et al. [4]. As EGF and gastrin are known in other contexts to be involved in activation of STAT3, it would be interesting to learn if STAT3-mediated *Ngn3* activation plays a role in the effects reported by Zhang et al. [6].

While these key recent findings connect extracellular signaling pathways to in vivo β-cell reprogramming of acinar and ductal cells through *Ngn3* activation, an impressive boost to the field of β-cell regeneration also comes from the discovery of the role of gamma-amino butyric acid (GABA) receptor signaling in the conversion of α cells to β cells. Two complementary papers published this year [8, 9] beautifully illustrate how knowledge acquired from genetic modification of *Arx* and *Pax4*, key transcription factors involved in α to β cell conversion, inspired the design of a high-throughput screen of small molecules to identify compounds that could either induce *Pax4* or inhibit *Arx* expression in α cell lines in vitro. This led to the identification of the artemisinin antimalarial drug class (for example, artemether) as hit compounds that induce the conversion of α to β cells through activation of GABA_A_ receptor signaling in α cells [9]. Indeed, either artemisinin treatment [9] or long-term administration of GABA [8] results in robust conversion of α cells to β cells both in vitro and in vivo. Strikingly, artemisinin or long-term GABA administration in vivo inhibits either *Arx* function or *Arx* expression, respectively, leading to induction of *Pax4* expression. Hence, both drugs promote β-cell neogenesis from α cells in vivo in zebrafish and rodent models of diabetes, as well as in human islets in vitro. GABA treatment acts via the GABA_A_ receptor in α cells, whereas artemisinins stabilize the neuronal assembly protein gephryin in this receptor, thus enhancing GABA_A_ receptor signaling, and consequently inhibiting glucagon secretion. This suggests that a reduced extracellular concentration of glucagon is required for α to β cell conversion mediated by GABA receptor signaling.

In all these regenerative scenarios (Fig. 1)—acinar to β cell, ductal to β cell, and α to β cell—the transition state involving *Ngn3* activation is necessary for the reprogramming to occur. Even though the GABA_A_-receptor-signaling-mediated α to β switch per se is independent of *Ngn3,* the conversion of endogenous α to β cells appears to trigger α-cell neogenesis from ductal cells by reactivation of *Ngn3*, such that knockdown of *Ngn3* in GABA-treated pancreas exhibits a reduction in α to β cell conversion [8]. Interestingly, in a recent study by Cheng and colleagues [10], a fasting-mimicking diet (FMD) in mice was shown to promote *Ngn3*-driven β-cell regeneration through the inhibition of PKA and mTOR kinases. Also in this study, exposure of healthy and T1D human islets to low-glucose and low-serum fasting*-*mimicking medium in vitro was found to significantly stimulate the secretion of insulin and to induce *Sox2* and *Ngn3* expression. This may suggest that medium or low glucose levels play an important role in pancreatic lineage reprogramming into β cells through the activation of *Ngn3*.

## Conclusions

These recent findings suggest that pharmacological treatment with several compounds or growth factors or presumably an FMD can promote plasticity and β-cell regeneration in the pancreas in animal models in vivo. The signaling pathways involved in such plasticity change depending on the lineage source of the reprogramming (acinar, ductal, or α cells). Although there are common requirements for all of them, such as the acquisition of a transitional progenitor-like state with the activation of *Ngn3*, extracellular glucose and glucagon levels might also play an important role. Thus, these recent findings open the door to potential new therapies to restore β-cell mass in diabetic patients.

## Abbreviations

FMD: Fasting-mimicking diet
MPC: Multipotent pancreatic progenitor cell
T1D: Type 1 diabetes
T2D: Type 2 diabetes
