# Erratum to: Pancreatic β-cell regeneration: advances in understanding the genes and signaling pathways involved

**DOI:** 10.1186/s13073-017-0445-x

**Published:** 2017-06-06

**Authors:** Solomon Afelik, Meritxell Rovira

**Affiliations:** 1 Division of Transplantation, Department of Surgery, University of Illinois at Chicago, 840 South Wood Street, CSB 920 (Rm 502), Chicago, IL 60612 USA; 2 Center of Regenerative Medicine in Barcelona (CMRB), Dr. Aiguader 88, 08003 Barcelona, Spain

## Erratum

Following the publication of of this article [1], it was brought to our attention that unfortunately an error was introduced during copyediting:

Due to the error, the heading of the article’s second main section incorrectly reads: “β cell regeneration from pancreatic acinar, ductal or β cell lineages requires *Ngn3* activation”.

The correct heading should instead read: “β cell regeneration from pancreatic acinar, ductal or α cell lineages requires *Ngn3* activation”.
